# Indications for hand and glove disinfection in Advanced Cardiovascular Life Support: A manikin simulation study

**DOI:** 10.3389/fmed.2022.1025449

**Published:** 2023-01-06

**Authors:** Stefan Bushuven, Joachim Bansbach, Michael Bentele, Stefanie Bentele, Bianka Gerber, Nicolas Reinoso-Schiller, Simone Scheithauer

**Affiliations:** ^1^Institute for Infection Control and Infection Prevention, Hegau-Jugendwerk Gailingen, Health Care Association District of Constance, Gailingen, Germany; ^2^Institute for Medical Education, University Hospital, LMU Munich, Munich, Germany; ^3^Department of Anesthesiology and Critical Care, Medical Center - University of Freiburg, Freiburg, Germany; ^4^Institute for Anaesthesiology, Intensive Care, Emergency Medicine and Pain Therapy, Hegau Bodensee Hospital, Singen, Germany; ^5^Training Center for Emergency Medicine (NOTIS e.V), Engen, Germany; ^6^Department of Emergency Medicine, University-Hospital Augsburg, University of Augsburg, Augsburg, Germany; ^7^Department of Infection Control and Infectious Diseases, University Medical Center Göttingen (UMG), Georg-August University Göttingen, Göttingen, Germany

**Keywords:** BLS (Basic Life Support), hand disinfection, infection prevention, CPR - cardiopulmonary resuscitation, life support, ACLS (Advanced Cardiovascular Life Support), glove disinfection, hospital acquire infection

## Abstract

**Background and aim:**

There are no investigations on hand hygiene during cardiopulmonary resuscitation (CPR), even though these patients are at high risk for healthcare-associated infections. We aimed to evaluate the number of indicated hand hygiene per CPR case in general and the fraction that could be accomplished without delay for other life-saving techniques through standardized observations.

**Materials and methods:**

In 2022, we conducted Advanced Cardiovascular Life Support (ACLS) courses over 4 days, practicing 33 ACLS case vignettes with standard measurements of chest compression fractions and hand hygiene indications. A total of nine healthcare workers (six nurses and three physicians) participated.

**Results:**

A total of 33 training scenarios resulted in 613 indications for hand disinfection. Of these, 150 (24%) occurred before patient contact and 310 (51%) before aseptic activities. In 282 out of 310 (91%) indications, which have the highest impact on patient safety, the medication administrator was responsible; in 28 out of 310 (9%) indications, the airway manager was responsible. Depending on the scenario and assuming 15 s to be sufficient for alcoholic disinfection, 56–100% (mean 84.1%, SD ± 13.1%) of all indications could have been accomplished without delaying patient resuscitation. Percentages were lower for 30-s of exposure time.

**Conclusion:**

To the best of our knowledge, this is the first study investigating the feasibility of hand hygiene in a manikin CPR study. Even if the feasibility is overestimated due to the study setup, the fundamental conclusion is that a relevant part of the WHO indications for hand disinfection can be implemented without compromising quality in acute care, thus increasing the overall quality of patient care.

## Introduction

### Background/rationale

In Germany, ~84 of every 100,000 persons annually suffer an acute cardiac arrest requiring early cardiopulmonary resuscitation (CPR), activation of the emergency chain, Advanced Cardiovascular Life Support, transportation, and integrated critical care (1). Hospital-acquired infections (HAI), mainly device-associated bloodstream infections, urinary tract infections, and pneumonia, significantly impact the mortality and morbidity of these patients, especially in those with hypoxemic brain injury (2, 3). Proper hand hygiene, especially before aseptic procedures, can significantly reduce these infections (4), especially in critical care settings. Under the recognition of national recommendations, the overall objective is to accomplish 80% of all indicated hand disinfection (5, 6). These comprise five main indications according to the five moments of hand hygiene: before touching the patient (WHO-1), before clean/aseptic procedures (WHO-2), after body fluid exposure/risk (WHO-3), after touching a patient (WHO-4), and after touching the patient's surroundings (WHO-5) (4, 6).

Currently, there are no investigations on the significance of infection prevention and control (IPC) in out-of-hospital cardiac arrest (OHCA) or in-hospital cardiac arrest (IHCA). Learning material for rescue service staff considers hand hygiene to be “good medical practice” and partially shows hand disinfection in educational videos provided by the American Heart Association (AHA) (7, 8). However, the need for IPC and especially hand hygiene is poorly emphasized in educational material, despite the effect of hand hygiene on nosocomial infection in general and in the ICU is high and considered a cornerstone of patient safety (9–13).

In general, hand hygiene is not conducted consistently in emergency situations such as trauma resuscitation (14) or Advanced Cardiovascular Life Support (ACLS). These situations involve potentially hazardous invasive procedures under time pressure, such as intravenous or intraosseous catheter placement, medication preparation and administration, endotracheal intubation, endotracheal suctioning, thoracocentesis, and in some cases, mini-thoracotomy, pericardiocentesis, or even clamshell-thoracotomy (15). All of these interventions are aseptic clean procedures according to the WHO's moments of hand disinfection (4, 6), and it may appear that they can be sacrificed to save time because the immediate demand for life-saving procedures precludes the time-consuming hand or glove disinfection.

Survivors of sudden cardiac arrest who require critical care are susceptible to infections with devastating effects like sepsis. In addition, post-hypoxic brain tissue and its penumbra are most vulnerable to inflammation, and outcomes may be even worse with fever (16). Therefore, rational infection prevention should be an integral part of life support from the first patient contact.

**Figure 1:**
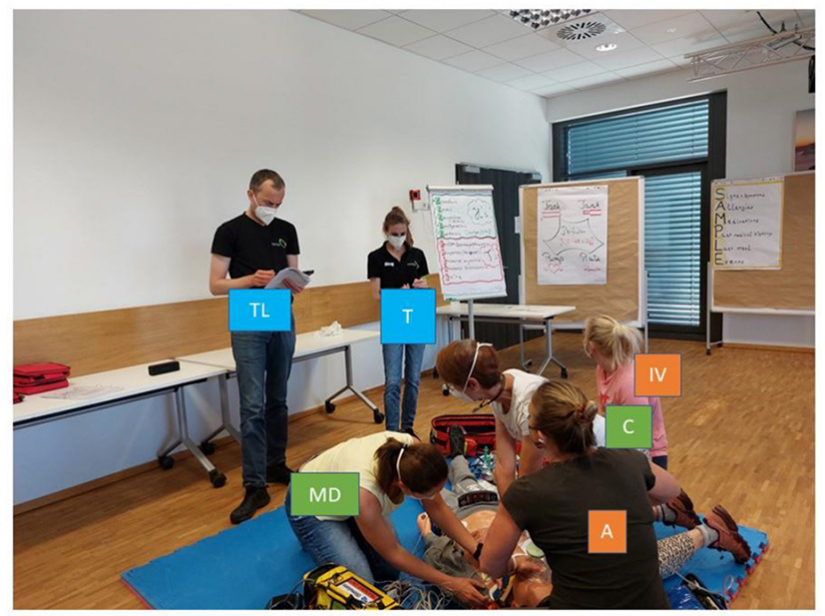
A prototypical ACLS training scenario with six members: the team leader (TL) and timekeeper (T) normally do not interact with the patient and do not perform invasive procedures. The compressor (C) and monitor/defibrillator (MD) may change roles and provide chest compressions to maintain cerebral and coronary perfusion. They also both typically do not perform invasive procedures. The monitor/defibrillation manager (MD) attaches electrodes to the patient's chest, analyses the ECG, and delivers shocks as indicated. The airway manager (A) ventilates with a bag valve mask, clears the airway if it is obstructed, and administers oxygen. If indicated, the airway manager places a supraglottic or endotracheal airway device. Hence, invasive procedures are sometimes performed by the airway manager or an assisting person (e.g., M or C), depending on the situation and crew resources. The medication administrator (IV) establishes intravenous or intraosseous access and prepares and administers medications according to the CPR or ROSC algorithm as identified and communicated by the team leader. The medication administrator is the person with the most expected invasive procedures and therefore the most hand hygiene indications. After each scenario, the roles were changed. It is noted that individuals are not wearing hospital clothing or personal protective equipment due to the training settings. N95 respirators were worn due to the COVID-19 pandemic. All depicted persons gave written informed consent for photography.

Healthcare providers are trained in CPR proficiency according to international or national recommendations for resuscitation provided by the International Liaison Committee on Resuscitation (ILCOR). The most widely used training concepts include scenarios provided by the Advanced Cardiovascular Life Support (ACLS) and Pediatric Advanced Life Support (PALS) programs that are available all around the world (8). ACLS and PALS primarily consist of standardized simulation-based learning for groups of six (about 4–7 persons) on manikins. These individuals share roles and responsibilities for different CPR actions (see Figure 1).

1. TL – Team leader
2. Guides and supervises the team
3. A – Airway manager
4. Conducts bag mask ventilation, oxygen supplementation, airway or tube suctioning, and intubation
5. C – Compressor
6. Completes the BLS-Check and provides chest compressions
7. MD – Monitor/Defibrillation
8. Attaches electrodes and the monitor, delivers electrotherapy, and supervises the quality of chest compressions and ventilations
9. T – Timekeeper
10. Records the amount of time taken and documents the CPR
11. IV – Medication administrator
12. Establishes venous or intraosseous access and prepares and administers IV/IO medication

**Figure 2:**
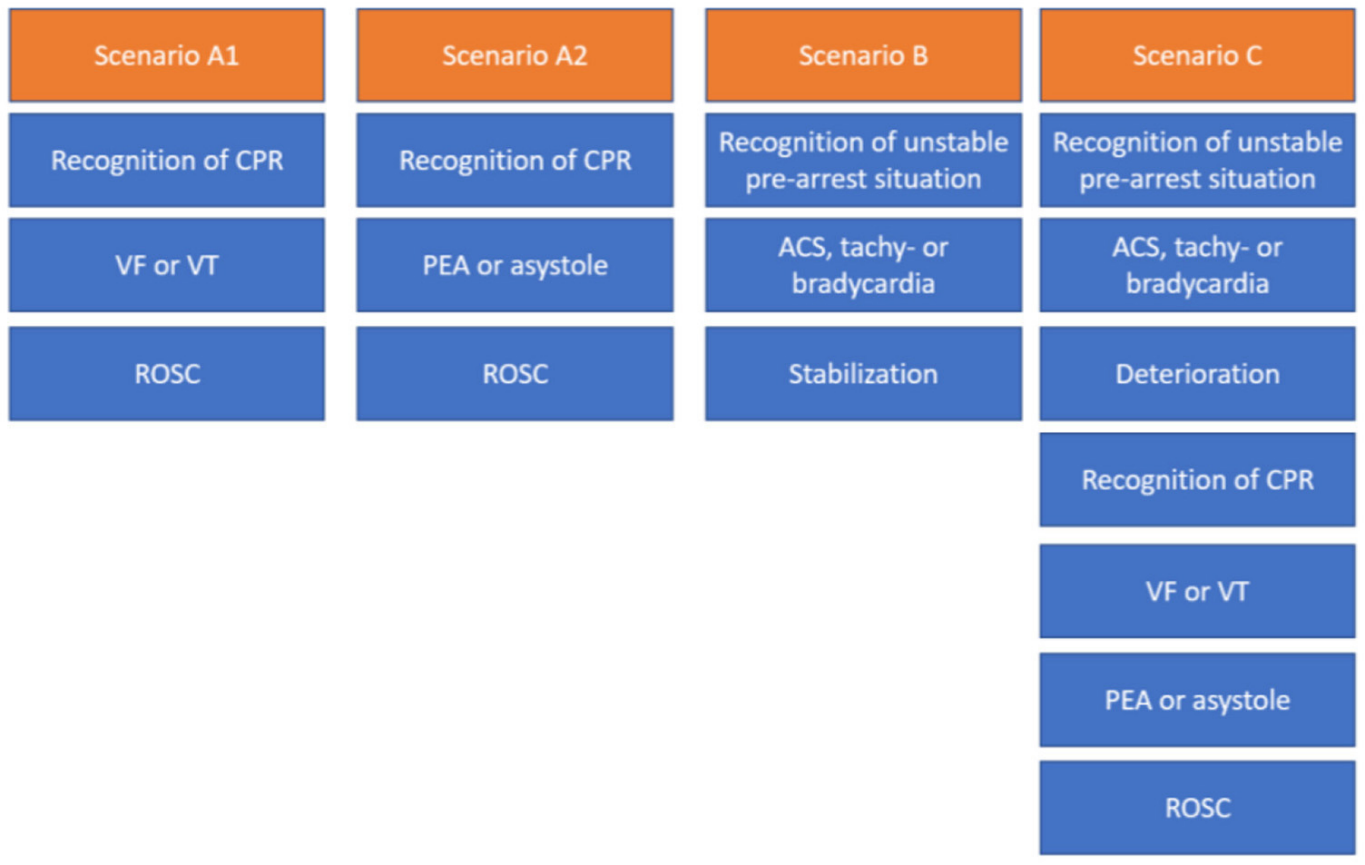
Different scenario types in ACLS courses typically last about 10–25 min each, including the briefing and debriefing. CA, cardiopulmonary arrest; VF, ventricular fibrillation; pVT, pulseless ventricular tachycardia; PEA, pulseless electrical activity; ROSC, return of spontaneous circulation; ACS, acute coronary syndrome.

During these courses, team performance and communication are evaluated and reflected on while debriefing. The training is conducted by certified course instructors who guide the trainees through different prototypical case vignettes of pre-arrest, arrest, and combined scenarios (17), as shown in Figure 2.

### Objective

This observational study aimed to evaluate how many indications are followed for hand hygiene per CPR and according to the five moments of hand hygiene and per case occurrence, how many of these indicated hand disinfections could be accomplished without delaying patient resuscitation.

We hypothesized that more than 80% of all WHO moments indicating the need for hand hygiene could be performed without losing time for other life-saving actions in the ACLS algorithms.

## Methods

### Study design and setting

In 2022, we held ACLS courses over 4 days with 4–5 providers practicing 33 ACLS case vignettes in an ACLS course (2 days), an ACLS refresher course (1 day), and an ACLS course for experienced providers (1 day). The case vignettes (see Tables 1, 2 and Figure 2) consisted of either vignette Type A1, A2, B, or C. In the ACLS courses, provided by NOTIS e.V (Notfallmedizinisches Trainingszentrum in Singen, a registered association), we used an AmbuMan Airway Manikin (Ambu GmbH, Bad Nauheim, Germany) and an ALSi Monitor (iSimulate, 3b Scientific GmbH, Hamburg, Germany). In addition, we used real-life equipment typically available in German hospitals and emergency medical services. This included a bag valve mask with oxygen supply, a backpack with ampules, sterile syringes, suction, an IO access device (Arrow EZIO, Teleflex, Morrisville, USA), IV catheters (Vasofix Safety, B. Braun, Melsungen, Germany), infusion bags, and documentation cards.

**Table 1:** Scenarios in ACLS (Advanced Cardiovascular Life Support Course), in ACLS-R (ACLS-refresher course for providers with preceding certification in ACLS, and ACLS-EP (ACLS for experienced providers with more complex or rare cases of cardiac arrest and peri-arrest).

**No**	**Scenario description**	**Group**	**Team**	**Type**	**WHO2 count**	**WHO-2 realisable**	**Initial rhythm**	**Initial rhythm detection time**	**Seconds to first i.v-drug indicated**
1	Lifeless person at the train station, asystole	ACLS	5	A	10	50.00%	ASY	30	30
2	ED Patient with acute deterioration and ventricular fibrillation	ACLS	5	A	7	100.00%	VT/VF	62	180
3	Unresponsive person at the lakes, hypoglycaemia, ventricular fibrillation	ACLS	5	A	14	78.57%	VT/VF	50	180
4	Obstetric Ward, collapsed visitor, ventricular fibrillation	ACLS	5	A	6	66.67%	VT/VF	148	180
5	Geriatric patient after fracture of the femoral neck, deterioration after aspiration, pulseless ventricular tachycardia	ACLS	5	A	10	80.00%	VT/VF	55	180
6	Oncological patient, asystole	ACLS	5	A	15	26.67%	ASY	60	60
7	Post ACS patient at the rehabilitation hospital, initially stable bradycardia, i.v already placed, later deterioration	ACLS	5	B	6	33.33%	BRADY	30	30
8	ED patient with ACS, instable bradycardia, i.v already placed	ACLS	5	B	4	100.00%	BRADY	105	105
9	Patient in the recovery room after cholecystectomy, initially stable supraventricular tachycardia; i.v already placed	ACLS	5	B	6	100.00%	SVT	18	
10	Elderly patient at the traumatological ward, fracture of the femoral neck, irregular instable supraventricular tachycardia, i.v. in place	ACLS	5	B	7	57.14%	SVT	27	
11	Unconscious patient in the park with acute coronary syndrome, instable bradycardia	ACLS	5	B	9	100.00%	BRADY	61	61
12	Patient with STEMI in the emergency department, instable broad complex tachycardia, then ventricular fibrillation and later asystole i.v. in place	ACLS	5	C	7	57.14%	BRADY	40	40
13	Old lady with abdominal pain due to NSTEMI, bradycardia, later ventricular fibrillation	ACLS	5	C	9	44.44%	BRADY	36	36
14	Patient with alcohol intoxication at a parking garage, instable superventricular tachycardia later ventricular fibrillation and asystole	ACLS	5	C	5	100.00%	SVT	67	
15	Mass casualty incident / overcrowding in the emergency department, patient with STEMI and instable bradycardia, later pulseless ventricular tachycardia and pulseless electrical activity, i.v. in place	ACLS	5	C	9	55.56%	BRADY	40	40
16	Dialysis patient with acute coronary syndrome and hyperkalaemia, instable bradycardia, ventricular fibrillation, later asystole	ACLS	5	C	11	54,55%	BRADY	40	40
17	Emergency department patient with STEMI, instable bradycardia, later ventricular fibrillation and asystole, i.v. in place	ACLS	5	C	11	63,64%	BRADY	28	28
18	Patient at the cardiological ward after percutaneous coronary intervention, STEMI, instable supraventricular tachycardia, then ventricular fibrillation and later pulseless electrical activity	ACLS	5	C	5	100.00%	SVT	27	
19	Emergency department patient with acute hemiparesis, suspected aortic dissection, bradycardia, ventricular fibrillation and later asystole, i.v.in place	ACLS	5	C	10	30.00%	BRADY	34	34
20	Patient at the urological ward, bradycardia, later ventricular fibrillation and asystole	ACLS	5	C	10	80.00%	BRADY	63	63
21	Patient at the orthopaedic ward after surgery, bradycardia, ventricular fibrillation and later pulseless electrical activity	ACLS	5	C	8	37.50%	BRADY	30	30
22	Lay rescuer CPR at the airport after two shocks by AED, persistent ventricular fibrillation	ACLS-R	4	C	8	37.50%	VT/VF	20	
23	Patient with instable bradycardia with AV-Block III° with conversion to ventricular fibrillation and later pulseless electrical activity	ACLS-R	4	C	13	76.92%	BRADY	64	64
24	Young athlete with initially stable supraventricular tachycardia and ventricular fibrillation after adenosine cardioversion (Wolff-Parkinson-White-Syndrome)	ACLS-R	4	C	10	100.00%	SVT	212	212
25	Elderly patient in an ice cream café, bradycardia due to NSTEMI, later ventricular fibrillation and pulseless electrical activity	ACLS-R	4	C	19	73.68%	BRADY	58	58
26	Retirement Home, collapsed nurse with instable bradycardia and later ventricular fibrillation	ACLS-R	4	C	12	41.67%	BRADY	30	30
27	ICU-patient after robotic prostatectomy, acute coronary syndrome with supraventricular tachycardia, later pulseless ventricular tachycardia, and asystole	ACLS-R	4	C	8	100.00%	SVT	40	
28	Elderly women in long term caring home, AV-Block III degree, later ventricular fibrillation, and pulseless electrical activity	ACLS-R	4	C	14	64,29%	BRADY	45	45
29	Syncopal women at the supermarket, initially stable supraventricular tachycardia and later ventricular fibrillation	ACLS-R	4	C	8	100,00%	SVT	32	
30	Pregnant in 22nd gestational week, thrombosis with obstructive shock due to pulmonary embolus, supraventricular tachycardia and pulseless electrical activity, emergency c-section	ACLS-EP	4	C	14	85,71%	SVT	45	
31	Young women with “herbal” intoxication and initially stable supraventricular tachycardia and later ventricular fibrillation	ACLS-EP	4	C	8	100,00%	SVT	50	
32	Electricity worker working at the ceiling installations of a private swimming pool, high level fall after accidental electric shock from a ladder into the pool, traumatic brain injury, drowning, pulseless electrical activity	ACLS-EP	4	C	9	22.22%	ASY	25	25
33	Lightning strike at a music festival, mass casualty incident with 20 persons and two persons in cardiac arrest, pulseless electrical activity	ACLS-EP	4	C	8	62,50%	ASY	40	40

“Initial rhythm detection time” was the time from arrival of the crew to recognition of the underlying rhythm. “Seconds to first i.e., drug indicated” was the time after assessing the patient until the first i.e., medication was necessary according to the algorithms. Missed values in some scenarios were those with a variable time of first indication, e.g., in initial stable conditions leading to CPR later, ongoing therapy by “others” (team arrives during ongoing therapy), pure pre-arrest scenarios (without CPR), or difficult conditions with the need for evacuation/transportation of the victim.

**Table 2:** Example of a prototypical case with possible indications for hand hygiene.

**Timeline**	**Situation /Algorithm**	**TL**	**TK**	**C**	**A**	**M**	**IV**
−3 min	Victim lies collapsed on the floor of the hospital hallway. Alarm on collapse, bystander BLS						
	ACLS Team informed	**WHO-1** Assignes roles and responsibilities	**WHO-1** ^*^	**WHO-1** ^*^	**WHO-1** ^*^	**WHO-1** ^*^	**WHO-1** ^*^
+ 0 m 0 s		ACLS Team approaches & Safety check					
+0 m 15 s	BLS Algorithm		Starts recording	BLS check, Tap & Shout and check for pulse and breathing			
+0 m 25 s	BLS Algorithm			Recognition of Arrest Start thorax compression			
+0 m 30 s	Arrest Algorithm				Start ventilation with mask - bag device and oxygene supply	Start ECG electrode placement	WHO-2^**^ Prepare IV-Access
+0 m 40 s	VF/VT Algorithm	Communicates Algorithm				Recognition of VF Loading Defibrillator	
+0 m 50 s	VF/VT Algorithm			Re-Start CPR after shock	Re-Start ventilation after shock	Clear Team, Defibrillation	
+1 m 30 s	VF/VT Algorithm						WHO-2^*^ Placement of i.v. Access, exposure to blood possible
+2 m 0 s	VF/VT Algorithm						WHO-2/3^*^ Preparation of Infusion bag
+2 m 50 s	VF/VT Algorithm					2nd ECG Check recognition of VF loading defibrillator	
	VF/VT Algorithm			Re-Start CPR after shock	Re-Start ventilation after shock	Clear Team, SHOCK	
+3m 0 s	VF/VT Algorithm						WHO-2^*^ Connection of crystalloid with venous access, possible contamination with blood
	VF/VT Algorithm						WHO-2/3^**^ [Preparation of 1mg epinephrine]
+4m 0 s	VF/VT Algorithm						WHO-2** Administration of epinephrine
+4m 50 s	VF/VT Algorithm					2nd ECG Check Recognition of VF Loading defibrillator	WHO-2/3** [Prepare 2nd dose epinephrine]
	VF/VT Algorithm			Re-Start CPR after shock	Re-Start ventilation after shock	Clear Team, shock	
+5m 20 s	VF/VT Algorithm						WHO-2** [Prepare 1^st^ dose Lidocaine or Amiodarone]
+6m 50 s	VF/VT Algorithm					3nd ECG Check Recognition of ROSC	
+7m 10 s	ROSC Algorithm	Communicates Algorithm		BLS Check Pulse, but unresponsive	Ventilation check	Apply 12-Lead ECG	
+7m 40 s	ROSC Algorithm STEMI Algorithm	Communicates Algorithm				ECG: Recognition of STEMI	WHO-2^*^ Start preparation of ASS i.v.
+8m 10 s	ROSC Algorithm STEMI Algorithm			Preparation of Intubation e.g. by “C” for “A”	Suctioning Airway in case of regurgitation WHO-3^*^		**WHO-2** Administration preparation of ASS i.v.
+9m 0 s	ROSC Algorithm STEMI Algorithm				Restarts Ventilation	WHO-2^*^ Fingertip puncture	WHO-2^*^ [Preparation of Heparine]
+10m 0 s	ROSC Algorithm STEMI Algorithm					Glucose measurement	WHO-2^*^ Administration of Heparine
+10m 30 s	ROSC Algorithm STEMI Algorithm			Intubation ready (ET-Tube with guidewire)			WHO-2^**^ [Preparation of hyponotic]
+11 m 0 s	ROSC Algorithm STEMI Algorithm				Preparation for Intubation		WHO-2^*^ Administration of hypnotic
+11 m 30 s	ROSC Algorithm STEMI Algorithm			Assist intubation	WHO 2^*^ Intubation attempt, suctioning airway	Recognize Monitor changes	
+11 m 45 s	ROSC Algorithm STEMI Algorithm			Fixation of the ET-Tube	WHO 2/3^**^ Apply mechanical ventilation and capnography		
	ROSC Algorithm STEMI Algorithm			Prepare Transport of patient	Prepare Transport of patient	Prepare Transport of patient	Prepare Transport of patient
+X	Handover to second Team (cathlab)	Communicates with cath lab crew					
	Team dismisses	WHO-5^*^	WHO-5^*^	WHO-3/4^*^	WHO-3/4^*^	WHO-3/4^*^	WHO-3/4^*^
	Structured Debriefing	Communicates with ACLS crew					

In this algorithm-based case and a team with six members, there are altogether 30 indications for hand hygiene that can be counted. In this theoretical “algorithm-based” scenario, we assumed that each i.v. medication preparation needs a separate hand disinfection before (which is realistic whenever the airway manager is involved in other interrupting actions). Errors and deviations from the algorithm are not considered in this table, and thus reality can further deviate from these estimations. Preparations with the possibility of prefilled syringes are placed in brackets. Time estimation was considered 15 s for hand disinfection. ^*^Feasible. ^**^Feasible for 15 s but not for 30-s hand disinfection.

### Participants

Recruited participants (*n* = 9) were ICU nurses and anesthesiologists from different institutions in southern Germany. All participants were informed about the observation and agreed to participate. Further instructions were not needed as participants were not expected to simulate or conduct hand hygiene or other IPC measures that would have deviated from the AHA course protocol. All participants rotated through the roles with different scenarios and were evaluated in the role of the team leader.

According to the Ethical Committee of the Physician Board Association of Baden-Württemberg, no ethical approval was needed. The data was obtained anonymously. The study protocol aligns with the Declaration of Helsinki and the German Physician Professional Code: there was no intervention in the personal, psychological, or somatic integrity of the participants, no data that could be retraced to a single person, and there was no data retrieved from patients.

### Variables and data sources

The primary variables consisted of the following:

a) The number of observed hand disinfection indications according to the WHO
b) The type of moment indicating hand disinfection (WHO 1–5)
c) The time from the indication of medical action to the *de facto* conduction of the action (“action time”)
d) The person responsible for hand disinfection according to the ACLS – roles
e) Type of CPR scenario and first identified heart rhythm

The secondary variables included arrest time and chest-compression-fraction (CCF– a surrogate parameter for CPR quality). CCF, arrest time (AT), and compression time (CT) were simultaneously measured using a stopwatch. CCF was calculated as the index of CT/AT.

Hand disinfection was “feasible” if the response time between the indication and the conduction thereof was at least 60 s, which includes 15 s of exposure time to the disinfectant (18). Furthermore, an additional 45 s for an aseptic procedure were granted according to the consensus of six specialists for CPR training.

The whole data was collected by the principal investigator as a certified ACLS instructor, specialist for infection control, and medical educator (single observer approach).

### Bias

We addressed the observer bias in this single-researcher approach by using prototypical case vignettes with easily reproducible choreography to maintain validity and reliability. One can question not using video recording but rather “observed” results. However, the prototypical cases are standardized internationally with a clearly defined structure, so we decided not to use videos because they could distract trainees and are not an integral part of certified AHA (American Heart Association) courses. Therefore, we combined the measurements of hand disinfection [which are used the same way in classical audits of hand disinfection (19)] with instructor-based observations (including measurements of CCF and team performance).

The performance bias could have occurred if there would have been any feedback on hand hygiene to the trainees and therefore improved performance in hand hygiene. However, at this point in the project, we did not provide any feedback on hand hygiene to limit this bias and maintain the ACLS training structure.

### Study size

We aimed for at least 30 ACLS training scenarios for better reproducibility and to rule out outlier scenarios.

### Statistical methods

We used descriptive statistics of the scenarios with standard measurements of CCF and hand hygiene indications. Statistics, including explorative data analyses and Mann-Whitney U-test, were conducted using Microsoft Excel (Microsoft Corporation, Redmond, USA), Addinsoft XL STAT (Addinsoft Inc., New York, USA), and IBM SPSS 27.0 (IBM Corporation, Armonk, USA).

## Results

### Participants and descriptive data

Overall, we examined 33 scenarios conducted in two ACLS courses with five and four participants, respectively, and guided by three certified instructors each. The participants were six nurses and three anesthesiologists eligible for ACLS courses according to the AHA. The instructors were physicians with an AHA instructor certification in ACLS (Advanced Cardiovascular Life Support) and ACLS-EP (ACLS for Experienced Providers, a course giving deeper insight into life support and special conditions, such as drowning, pregnancy, and intoxications). The cases presented during the course are shown in Table 1. Chest compression fractions ranged from 55.8 to 97.0 % (mean 81.2%), mainly depending on the scenario type (see Table 1) and learning progress.

### Main results

Overall, 613 indications for hand disinfection could be observed in the 33 scenarios. Of these indications, 150 occurred before touching a patient (WHO-1), 310 occurred before clean/aseptic procedures (WHO-2), three occurred after body fluid exposure risk (WHO-3), and 150 occurred after touching a patient (WHO-4) or after contact with the patient's surroundings (WHO-5) indications. WHO-1, WHO-4, and WHO-5 hand disinfection indications were considered appropriate to carry out before attending to the patient and after handover to other teams of the emergency chain. Due to the training setting on manikins, the WHO-3 indications varied depending on the case choreography (e.g., description of vomiting or dislocation of peripheral lines). The WHO-2 moments occurred most frequently and at different stages during the scenarios (e.g., IV medication administration or airway manipulation) and are therefore the most significant to analyze in detail.

Per one scenario, we detected between 14 and −27 WHO 1–5 indications (mean 18.6, SD 3.2) and between 4 and 19 WHO-2 indications (mean 9.0, SD 3.0). Depending on the scenario, 56–100% (mean 84.1%, SD = 13.1%) of all indications could have been accomplished without delaying patient resuscitation.

Of the 310 WHO-2 indications (before an aseptic procedure), 282 suggested the responsibility of the medication administrator (91.0%) and 28 of the airway manager (9.1%). For each CPR scenario, the medication administrator had to expect 4–17 WHO-2 indications and airway managers 0–2 WHO-2 indications. There were no WHO-2 indications detected for other team members.

**Figure 3:**
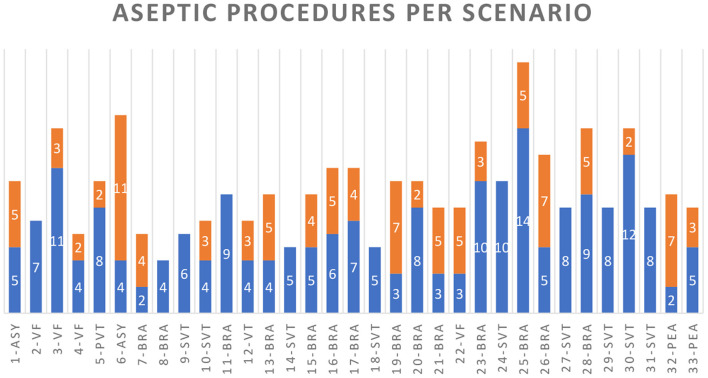
WHO-2 indications for cases 1–33 with the scenarios: asystole (ASY), ventricular fibrillation (VF), pulseless ventricular tachycardia (PVT), bradycardia (BRA), unstable VT with a pulse (VT), supraventricular tachycardia (SVT), and pulseless electrical activity (PEA). Blue columns indicate the hand or glove disinfection indications that were feasible and orange for those that were not.

For the medication administrator, 186 of 282 hand disinfections (66.0%) and for the airway manager, 22 of 28 (78.6%) hand disinfections would have been feasible without delay for patient care according to ACLS algorithms (see Figure 3). In scenarios with an immediate need for IV access (unstable bradycardia, asystole, and PEA), the count of unfeasible hand disinfection was significantly higher than in scenarios with subsequent indications for IV drug administration, such as supraventricular tachycardia (SVT) and ventricular tachycardia/ventricular fibrillation (pVT/VF) (*p* = 0.002). In contrast, the count of feasible hand disinfection did not significantly differ between these scenario types (*p* > 0.05). Scenario 22 (VF) was an exception, as lay rescuer CPR with two given shocks occurred before the high-performance team arrived, and therefore an early administration of epinephrine was required.

The feasible hand hygiene indications for the airway manager (or any person assisting) include the preparation of the endotracheal tube with a guidewire, laryngoscopy, endotracheal intubation, and the ventilator setup for a patient not awakening after ROSC with sufficient time for hand or glove disinfection while the compressor repeats the BLS check according to the ROSC algorithm. On the contrary, a difficult airway during bag-mask ventilation was simulated in some cases (“cannot ventilate” – situation), which, to our interpretation, shows no opportunity for hand hygiene without delay for the patient's airway safety.

Regarding the medication administrator, participants placed an IV or IO access early with preparing and connecting a crystalloid infusion. However, as the identification of the heart rhythm determines the algorithm, IV access and administration of medications are necessary as soon as the algorithm is clear. In most ACLS cases, the approach to the patient, BLS check, attachment of the monitor, and correct identification of the heart rhythm ranged between 18 and 212 s (mean 51.85 s, SD 37.7). In CPR scenarios with the VT/VF algorithm, the first IV medication should be considered after the second shock (after 240 s of CPR/2 CPR cycles), providing enough time for IV drug preparation, IV access, and hand disinfection. The asystole or pulseless electrical activity algorithm recommends giving 1 mg of epinephrine as fast as possible. It should, therefore, ideally be administered directly after recognition of the rhythm. All four cases with initial asystole or PEA (scenarios 1, 6, 32, and 33) showed low feasibility of hand disinfection in WHO-2 indications in the first minutes (hand hygiene possible in 70.0%, 26.7%, 22.2%, and 62.5%). In contrast, the megacode scenarios with sequential asystole or PEA after VT/VF showed higher feasibility as the first i.v. medication is conducted after the second shock: after the first minutes of the peri-arrest or arrest algorithms, nearly all indications for the medication administrator showed to be foreseeable; thus, hand hygiene was feasible. These “late” indications included the repetitive administration of epinephrine (every 3–5 min), amiodarone, or lidocaine (after the third shock); the use of ACS medication after ROSC in ACS and peri-arrest scenarios (acetylsalicylic acid, heparin, rt-PA, morphine, hypnotics, and muscle relaxants); and measurements of blood sugar.

## Discussion

### Key results

To the best of our knowledge, this is the first study investigating the feasibility of hand hygiene in a manikin CPR study. In this study, we demonstrated that hand or glove disinfection is indicated repeatedly in prototypical arrest and peri-arrest scenarios. Approximately, 90% of all hand hygiene indications and, in many cases, more than 80% of WHO-2 indications are achievable during CPR without delay for resuscitation. That would align with the WHO recommendation of at least an 80% compliance rate (4), even in acute cardiac arrest scenarios. A total of 90% of the highly significant WHO-2 indications had to be accomplished by the role of the medication administrator and about 10% by the airway manager. Lower rates of realizable hand hygiene were detected in primary cardiac arrest scenarios with asystole or PEA and unstable bradycardia needing early drug administration.

### Limitations

This study has several limitations. First, this is a manikin study under ideal resuscitation conditions, with the likelihood of rapid IV access and a steep learning curve for the participants. The latter is shown by improving CCF rates and reducing the time to identify the first rhythm. These ideal conditions are not transferable to reality, where limited resources, different competencies, an additional need for team setup, and patient and environmental obstacles (e.g., difficult clothing, delayed rhythm identification, or difficult IV access) are common challenges. However, ACLS courses are widely acknowledged to effectively prepare staff for emergency situations (20–22), are satisfactory to participants (23), and can be considered a worldwide standard. Even if the feasibility is overestimated, the basic observation and statement remain true: a relevant part of the WHO indications could be implemented without delaying acute care, and thus, the overall quality of patient care could be increased.

Second, the selection bias must be mentioned as we examined 33 scenarios with 9 participants. From our viewpoint, the number of participants using highly standardized scenarios and algorithms does not play a significant role. If we had conducted the study with a new crew for each scenario and assumed adherence to AHA algorithms and choreographed scenarios, we would not expect significant changes to the number of hand hygiene indications, as these are mainly dependent on the scenarios. However, this hypothesis could be examined further, especially for errors in algorithms that may “produce” more or less indications. On the contrary, with succeeding simulation studies focusing on real-life hand hygiene protocol adherence, the number of participants would play a significant role as adherence is individually different and vulnerable to psychological effects (24, 25). In addition, other life support courses should be taken into account, such as PALS, ATLS, PHTLS, or ACiLS (26, 27) scenarios, to determine whether the number and opportunities for hand disinfection differ from ACLS scenarios. Consequently, real-time observations in real cases should clarify further differences between simulated and realistic cases (14).

Third, it is possible that the WHO-3 moments were under measured. These would have depended on the choreography of each case, which was not considered in this setting. Empirically, these indications may be relevant for all team members after contact with the patient's blood, esophageal regurgitation, vomiting, and respiratory secretions. Third, our study did not use video recording or other technically supported identification of WHO indications, possibly leading to observer bias. However, this presents an opportunity for further research with video-recorded observations, which add further data and enhance the validity of our findings.

A further limitation is that hand hygiene and the use of PSA were only indicated but not simulated. However, as a first approach, we decided not to alter the AHA course protocols for implementing PSA and hand hygiene. Our results show that hand hygiene could be implemented in several cases so that we are focusing on the realistic feasibility of the use of PSA and glove disinfection in a BLS follow-up study of our working group. Regarding observer bias, the use of videotaping or multiple observers in further examinations or real cases may strengthen test reliability.

### Interpretation and generalizability

Our study shows that, concerning hand hygiene, two roles of the CPR team must be focused on: the medication administrator and the airway manager. The other roles had just two hand hygiene indications: when arriving at the scene and after handover (WHO-1 and -4 or -5). These can be considered feasible in all cases.

The airway manager (or an assisting person) must carry out bag-mask ventilation or intubation after ROSC in unconscious patients. After ROSC, the need for a 12-lead-ECG and additional medication takes some time. Therefore, the airway manager and assisting crewmembers to have a foreseeable time window to prepare and conduct endotracheal intubation after hand hygiene (which is relevant long-term regarding the risks for nosocomial pneumonia).

For medication administrators, this is more complicated, as they have many tasks when arriving at the scene: place and open their medication backpack or trolley on the ground or a table, apply a tourniquet to the patient's arm, identify a puncturable vein, apply skin disinfection, unpack the IV set, perform hand disinfection, puncture the vein, secure the IV cannula, prepare a crystalloid infusion or saline syringe, connect the infusion bag, and prepare and administer IV medications. Especially in cases where there is a need for early drug administration (unstable bradycardia, asystole, PEA), this could be difficult. More time is available for the medication administrator to safely prepare and administer medications later in all algorithms or with a previously placed IV cannula.

It must be mentioned that most resuscitation teams might indicate the need for IV access according to the situation (a person in distress) and not the diagnosis made by examination and monitoring (“every emergency patient needs IV access as soon as possible”). This point is debatable, especially in peri-arrest emergencies when a greater focus should be placed on history-taking. This controversy should be considered in further investigations. For this study, we considered IV access indicated by the diagnosis, not by the situation.

Aside from using time-saving glove disinfection (28) without the problem of “wet hands in new gloves,” it might be possible to optimize hand hygiene for the medication administrator by providing prefilled syringes. This might apply to sodium chloride (for an IV push dose), epinephrine, lidocaine, amiodarone, and atropine. In a classical VF/VT scenario of 20 min CPR time, this could reduce the number of indications from 12 (Five dosages of epinephrine, two dosages of amiodarone) to 5. This reduction in medication preparation time can free up the medication administrator to fulfill other tasks, especially in a CPR setting with combined roles due to a staff shortage. In addition to the benefits of aseptic preparation, prefilled syringes might be preferable for correct dosing (29), fewer errors in selecting the correct drug (30), finding the correct doses (especially in pediatrics) (31), and reducing the risk of needle stick injuries when using needles for preparation.

Concerning the time for hand disinfection, we allowed 15 s for hand disinfection as this is suitable for the reduction of bacterial contamination of hands or gloves, which is more relevant for patients' nosocomial infections than viruses transmitted by contact. (18). Considering 30 s for hand hygiene would lower the feasibility rates of hand hygiene in some cases, especially in asystole/PEA: in scenario 5, the rate of the feasible hand disinfections would be the same as with 15 s as in VT-algorithms there is enough time before the first administration of epinephrine and enough time to prepare further medication as the case progresses. In contrast, in case 7, the rate of feasible hand disinfection would drop from 100 to 33%. Regarding the prototypical scenario (see Table 2), it is likely that, especially when prefilled syringes are not available, there would be a significant drop in justifiable hand disinfections due to the time-consuming preparation times for i.v. medications. Consequently, aside from the need for further research on this topic, there is a need for consensus and recommendations by infection prevention and resuscitation experts as to whether 15 s would be sufficient in the special situation of resuscitation or not.

Next, we did not consider the whole spectrum of hand disinfection agents like ethanol, 1-propanol, or 3-propanol, or even the durability of gloves after disinfection (28). Furthermore, we did not consider the problem that most disinfection agents are not fully virucidal and need 60 s for efficacy (32). Under CPR conditions and as mentioned above, the virucidal spectra might be considered less significant for patients as they are mainly susceptible to bacterial contamination of devices leading to bloodstream infection and pneumonia. However, it is relevant to healthcare providers caring for a patient with a viral disease (e.g., COVID-19), creating the need for the CPR team to wear protective equipment (33).

Furthermore, hand disinfection under CPR conditions may be subjectively seen as dispensable due to the emergency setting (“necessity knows no rules” – in German: “*Not kennt kein Gebot*”). In general, the discussion about the study after the courses resulted in irritation and amusement among single participants. We all strongly agree that hand disinfection must not delay life-saving care. However, we could demonstrate that hand hygiene is feasible in most cases and should not be abandoned categorically or carelessly, especially as it might ruin the success of resuscitation after some days due to nosocomial infection, sepsis, and multiorgan dysfunction. To raise awareness among healthcare workers, the prevalence of device-associated “post-ROSC pneumonia” (that may be interpreted as aspiration pneumonia) and “post-ROSC blood stream infection” should be investigated further. In addition, the learning material and videos presented to course participants should outline the role of IPC and post-ROSC removal of not aseptically placed IV lines.

It has to be emphasized that these findings clearly indicate the need for more research on the feasibility of hand hygiene under CPR conditions and post-ROSC, we need practical strategies to lower barriers to accomplish it. As we mentioned above, prefilled syringes may reduce the number of indications and may lower the risks of contamination. The use of double gloves and glove disinfection may also reduce contaminations. Furthermore, BLS- and ALCS-crews need a safe opportunity for hand disinfection, e.g., by small and easily accessible disinfection bottles for belts or smock pockets and perhaps a single dispenser for the IV-Manager. In wards and medical facilities, the visuality of dispensers is a factor for its use (34). In addition, algorithm-based pauses in algorithms, like team-time-outs (“10 s for the next 10 min”) (35) may be evaluated to grant generic glove disinfection for the whole team. Large teams (that are seldom in OHCA, but may be more often in IHCA) could even further split the role of the “IV” into an “IV” and “IV-assistant” – with regard to overcrowding phenomena.

Finally, our data were obtained in scenarios created to test the ACLS candidates using different algorithms. These scenarios seldom occur in reality, which reduces generalizability: according to the German databank for resuscitation (36), in 2021, about 21% of 16,265 detected out-of-hospital-cardiac arrests (OHCA) showed an initial shockable rhythm, whereas the remaining OHCA are due to asystole and PEA. Hypothetically, with about 10 WHO-2 indications in every asystole and PEA case and only 7% adherence to IPC protocols (14), this would result in approximately 151,125 omitted hand disinfections. According to our four cases with low justifiability for hand hygiene in 22–70% of the cases, approximately between 33,550 and 105,878 indicated and feasible hand disinfections would have been omitted. However, lower rates in the majority of OHCA do not justify general abandonment of hand hygiene at all in any resuscitation attempt: bio-ethically (37) such a generalized abandonment would be questionable in terms of benevolence (provision of best survival conditions to the patient), non-maleficence (infection prevention and secondary brain damage), and justice [providing best chances for survival due to reduced mortality, appreciating the work of healthcare providers in the chain of survival, and limiting the economic burden of hospital-associated infections (38, 39)].

Overall, these findings indicate the need for rational use of IPC in CPR conditions whenever feasible. Therefore, further training, raising awareness among CPR providers, and improving the education material are necessary.

## Conclusion

In our manikin study, we demonstrated that most hygienic hand or glove disinfection indications were feasible using the 15-s hand disinfection approach. Furthermore, we were able to show that the medication administrator faced most of the indications, of which more than 80% could be conducted. The situations in which hand hygiene was not performed were mainly in unstable peri-arrest rhythms, asystole, and pulseless electrical activity. Further work should concentrate on real-life scenarios, the role of prefilled syringes, investigating the role of device-associated post-ROSC nosocomial diseases, and education to reduce possible narratives that hand disinfection is dispensable in emergency situations.

## Data availability statement

The raw data supporting the conclusions of this article will be made available by the authors, without undue reservation.

## Ethics statement

The studies involving human participants were reviewed and approved by the Ethical Commitee Physicians Association Baden Wurttemberg, Germany. Written informed consent for participation was not required for this study in accordance with the national legislation and the institutional requirements. Written informed consent was obtained from the individual(s) for the publication of any potentially identifiable images or data included in this article.

## Author contributions

SBu: conceptualization, recruitment, primary manuscript draft, funding, and ethics. JB: medical validation (ERC recommendations). MB and SBe: medical validation (AHA recommendations). BG: validation, primary draft, and secondary draft of the manuscript (native speaker). NR-S: psychological expertise. SiSc: design, method, and manuscript. All authors contributed to the article and approved the submitted‘version.
